# Influence of Spinopelvic Alignment on Pelvic Tilt after Total Hip Arthroplasty

**DOI:** 10.1111/os.12469

**Published:** 2019-05-31

**Authors:** Makoto Kanto, Keishi Maruo, Toshiya Tachibana, Shigeo Fukunishi, Shoji Nishio, Yu Takeda, Fumihiro Arizumi, Kazuki Kusuyama, Kazuya Kishima, Shinichi Yoshiya

**Affiliations:** ^1^ Department of Orthopaedic Surgery Hyogo College of Medicine Nishinomiya Japan; ^2^ Department of Orthopaedic Surgery Takarazuka City Hospital Japan

**Keywords:** Anterior pelvic plane, Change in pelvic tilt, Hip‐spine syndrome; Total hip arthroplasty

## Abstract

**Objective:**

To evaluate the impact of spinopelvic parameters and hip contracture on change in the pelvic tilt (PT) after Total hip arthroplasty (THA).

**Methods:**

One hundred patients (15 male and 85 female) who underwent THA were included in this prospective study. Radiographic data were obtained preoperatively and 1 year after THA. Radiographic parameters included sagittal anterior pelvic plane (APP), sagittal vertical axis (SVA), sacral slope (SS), pelvic inclination (PI), and lumbar lordosis angle (LL). The APP was defined as the angle between the anterior pelvic plane and the vertical plane. A positive value indicates pelvic retroversion. Postoperative changes in PT were divided into three groups: the PA group (pelvic anteversion, ΔAPP < −5°), the PR group (pelvic retroversion, ΔAPP > 5°), and the PT group (minimal change, ΔAPP ≤ ± 5°). The Kruskal–Wallis test and the Steel–Dwass test were used to compare the preoperative and postoperative spinopelvic parameters among the three groups. The Spearman's rank correlation coefficient was used to evaluate the correlation between ΔAPP and spinopelvic parameters.

**Results:**

Minimal change in pelvic tilt was observed in 59% of patients, while pelvic anteversion was observed in 16% of patients and pelvic retroversion was observed in 25% of patients. There were no significant changes in the spinopelvic parameters, including TK, LL, SVA, LL, SS, and APP after THA. The Femoral angle (FA) was significantly decreased after THA (*P* < 0.001). Preoperative APP was significantly more retroverted in the PA group than the PR group, and the PT group (6.8 ± 12.2, 0.2 ± 9.9, −8.3 ± 8.3, *P* < 0.001). Preoperative SS, PI‐LL, and PI were significantly smaller in the PA group than the PT group and the PR group. A significant negative correlation was identified between preoperative APP and ΔAPP (*r* = −0.418, *P* < 0.001).

**Conclusion:**

Approximately 60% of the patients did not have any marked change in PT after THA. Preoperative APP was the only predictive factor associated with marked anterior or posterior change in PT.

## Introduction

The accurate positioning of the acetabular cup in total hip arthroplasty (THA) is important to optimize functional outcomes and to reduce the incidence of complications, including dislocation, impingement, and polyethylene wear^1^, ^2^, ^3^, ^4^. The authors of the current study have previously reported on the accuracy of acetabular cup orientation using a CT‐free navigation system^5^, ^6^. Cup inclination and anteversion angles were targeted at 35°–45° and 15°–25°^6^. Cup orientation is affected by the pelvic tilt (PT), and several studies have demonstrated the postural change in PT between sitting, supine and standing position^7^, ^8^, ^9^. Postoperative change in PT is important for accurate preoperative planning of acetabular cup angles. The effect of PT on acetabular cup anteversion has been reported to be an approximately 0.7° increase in anteversion for each degree of posterior PT^10^. Recently, sagittal spinopelvic alignment has been associated with health‐related quality of life (HRQOL)^11^. PT has been reported as a key parameter to maintain the sagittal balance which affects HRQOL^12^, ^13^. The total average change in PT after THA has been reported to be generally less than 10°, which is relatively small^14^, ^15^, ^16^, ^17^. The prediction of postoperative change in pelvic tilt after THA, especially anterior or posterior change, is important. However, there are not many reports about the correlation between anterior or posterior change in PT and spinopelvic parameters. The purpose of the present study was to evaluate: (i) how much anterior or posterior change in pelvic tilt was present after THA; and (ii) the correlation between anterior or posterior change in PT and spinopelvic parameters after THA.

## Methods

### *Patient Population*

The inclusion criteria were as follows: (i) patients with hip disease who were treated with THA between 2012 and 2014 at our hospital; (ii) preoperative and postoperative comparison of radiographic data was available. The exclusion criteria were as follows: (i) previous surgery for lumbar spine; (ii) previous infection; (iii) pathological fracture of the hip joint. This study was approved by our institutional review board.

A total of 100 patients (15 male, 85 female) met our inclusion criteria. The mean age at surgery was 61 ± 15 years. Preoperative diagnoses included primary osteoarthritis (OA) in 25 hips, developmental dysplasia of the hip (DDH) in 56 hips, osteonecrosis of the femoral head in 14 hips, and rheumatoid arthritis in 5 hips.

### *Surgical Procedure*

All surgeries were performed with a modified Hardinge approach in a lateral position using an image‐free navigation system to determine cup and stem alignment. Acetabular components differed according to the patient functional requirements and the surgeons’ preference. All hips were implanted with a cementless cup (Plasma cup B, B. Braun‐Aesculap, Tuttlingen, Germany) and a cementless stem (Bicontact, B. Braun‐Aesculap, Tuttlingen, Germany). Cup inclination and anteversion angle were targeted at 35° to 45° and 15° to 25°, respectively^18^. Their safe zones and formula provide a larger range of motion (ROM). In addition, surgeons considered combined orientation of cup anteversion and stem antetorsion. The concept of combined anteversion was proposed, using parameters to assess the optimal prosthetic alignment^4^.

### *Radiographic Evaluation*

Patients were asked to stand in a relaxed upright posture with their hip and knees as fully extended as possible. Angular measurements of sagittal pelvic and spinal parameters were performed on plain lateral X‐rays of the spine, including the pelvis, the femoral heads, and the upper part of the femoral diaphysis. Radiographic data were obtained preoperatively and 1 year after THA.

### *Radiographic Parameters*

Lumbar lordosis (LL): The angle between the cranial end plate of L_1_ and the cranial end plate of S_1_.

Thoracic kyphosis (TK): The angle between the cranial endplate of T_5_ and the caudal end plate of T_12_.

Pelvic incidence (PI): The angle between the line perpendicular to the middle of the cranial sacral endplate and the line joining the middle of the cranial sacral endplate to the center of the femoral head axis. The PI is the key parameter for determining the spinal balance.

Sacral slope (SS): The angle between the sacral endplate and the horizontal plane.

Femoral angle (FA): The FA is the angle between the vertical line and the femoral axis.

Sagittal vertical axis (SVA): Distance from the plumb line from the center of the C_7_ to the posterior edge of the upper sacral endplate.

Anterior pelvic plane angle (APP): The APP is the value of the angle between the vertical and the anterior pelvic plane.

### *Change in the Anterior Pelvic Plane*

**Figure 1:**
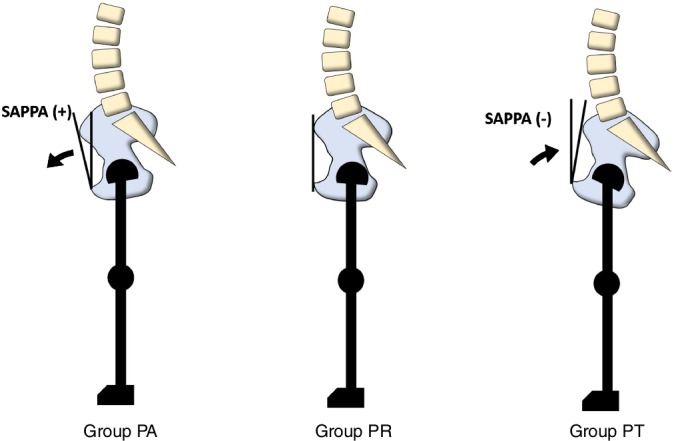
Change in pelvic tilt after total hip arthroplasty (THA). Group PA: Anterior pelvic tilt more than 5° after THA; Group PR: change in pelvic tilt within 5° after THA; Group PT, posterior pelvic tilt more than 5° after THA. A positive value reflected a pelvic anteversion.

A positive value reflected a pelvic anteversion, and a negative value reflected a pelvic retroversion (Fig. 1). Postoperative change in the APP (ΔAPP) was defined as the difference between preoperative and 1‐year postoperative values. The ΔAPP was categorized into three groups: (i) pelvic anteversion (PA group): ΔAPP > 5°; (ii) pelvic retroversion (PR group): ΔAPP < −5°; and (iii) minimal change in pelvic tilt (PT group): ΔAPP ≤ ±5°.

### *Statistical Analysis*

Continuous variables are presented as means ± SD with ranges, and categorical variables are presented as frequencies and percentages. Statistical analyses were performed using SPSS v19.0.1 (SPSS, Chicago IL, US). The Wilcoxon rank sum test was used to compare preoperative and postoperative radiographic parameters. The Kruskal–Wallis test and the Steel–Dwass test were used to compare the spinopelvic parameters among the three groups. The Spearman's rank correlation coefficient was used to evaluate the correlation between ΔAPP and spinopelvic parameters. Statistical significance was predetermined for *P*‐values < 0.05.

## Results

### *Measurement of Pelvic Tilt*

**Figure 2:**
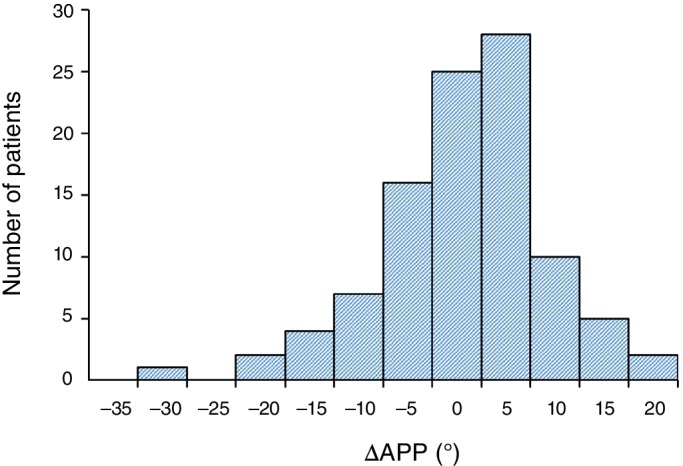
Distribution of ΔAPP after total hip arthroplasty (THA). Minimal change in pelvic tilt was observed in 59% of patients at 1‐year postoperatively.

Minimal change in PT (PT group) was observed in 59% (59/100) of patients at 1‐year postoperatively, while pelvic anteversion (PA group) was observed in 16% (16/100) of patients and pelvic retroversion (PR group) was observed in 25% (25/100) of patients. There was no significant difference between preoperative APP and postoperative APP at 1‐year follow‐up (Table 1). The distribution of ΔAPP after THA is shown in Fig. 2. A total of 81% of patients had ΔAPP ≤ 10°.

**Table 1:** Change in spinopelvic parameters after total hip arthroplasty

Variables	Preoperative	Postoperative	*P*‐value
FA (°)	9.2 ± 5.6	7.5 ± 4.5	<0.001*
LL (°)	46.6 ± 14.3	45.2 ± 14.9	0.289
TK (°)	25.9 ± 10.9	25.7 ± 11.9	0.836
APP (°)	0.1 ± 11.0	−0.1 ± 11.0	0.219
SVA (°)	21.0 ± 36.6	23.2 ± 36.0	0.396
SS (°)	38.4 ± 9.7	37 ± 9.9	0.003*

APP, anterior pelvic plane; FA, femoral angle; LL, lumbar lordosis; SS, sacral slope; SVA, sagittal vertical axis; TK, thoracic kyphosis.

* Statistically significant (*P* < 0.05).

### *Change in the Spinopelvic Parameters*

There were no significant differences between preoperative and postoperative LL, TK, and SVA. The FA (9.2° ± 5.6° *vs* 7.5° ± 4.5°, *P* < 0.001) and SS (38.4° ± 9.7° *vs* 37° ± 9.9°, *P* = 0.003) significantly decreased postoperatively. (Table 1).

### *Comparison of Preoperative Spinopelvic Parameters*

Preoperative APP was significantly more retroverted in the PA group than the PT group and the PR group. In contrast, preoperative APP was significantly more anteverted in the PR group than in the PT group and the PA group (Table 2). Preoperative SS (30.9° ± 9.5°, 40.6° ± 9.7°, 38.2° ± 7.5°, *P* = 0.003) and PI (35.9° ± 20.1°, 47.7° ± 17.2°, 46.8° ± 12.4°, *P* = 0.036) were significantly smaller in the PA group than in the PT group and the PR group. There was no significant difference among the three groups in preoperative parameters, including FA, LL, TK, and SVA. (Table 2).

**Table 2:** Comparison of preoperative spinopelvic parameters among three groups

Variables	PA group	PT group	PR group	*P*
Preoperative FA (°)	8.1 ± 5.7	9.6 ± 6.3	8.7 ± 3.4	0.653
Preoperative LL (°)	44.2 ± 16.5	47.5 ± 15.0	46.0 ± 11.1	0.638
Preoperative TK (°)	30.6 ± 13.3	24.9 ± 10.0	25.1 ± 11.1	0.519
Preoperative APP (°)	−6.8 ± 12.2	−0.2 ± 9.9	8.3 ± 8.3	<0.001*
Preoperative SVA (°)	8.4 ± 33.9	22.7 ± 37.8	25.1 ± 34.7	0.144
Preoperative SS (°)	30.9 ± 9.5	40.6 ± 9.7	38.2 ± 7.5	0.003*
Preoperative PI (°)	35.9 ± 20.1	47.7 ± 17.2	46.8 ± 12.4	0.036*
Preoperative PI‐LL (°)	−8.3 ± 27.7	0.3 ± 20.1	0.8 ± 16.4	0.042*

APP, anterior pelvic plane; FA, femoral angle; LL, lumbar lordosis; PI, pelvic incidence; SS, sacral slope; SVA, sagittal vertical axis; TK, thoracic kyphosis.

* Statistically significant (*P* < 0.05).

### *Comparison of Change in the Spinopelvic Parameters*

There was no significant difference between the groups, including ΔLL, ΔSS, and ΔSVA (Table 3); however, ΔTK (−5.6° ± 6.1°, 0.2° ± 6.7°, 1.8° ± 6.2°, *P* = 0.003) was significantly smaller in the PA group than in the PT group and the PR group (Table 3). Change in the APP was significantly more anteverted in the PA group (9.4° ± 3.7°) than in the PT group and the PR group. In contrast, change in the APP was significantly more retroverted in the PR group (−12.2° ± 6.3°) than in the PA group and the PT group (Table 3).

**Table 3:** Comparison of change in spinopelvic parameters among three groups

Variables	PA group	PT group	PR group	*P*
ΔLL (°)	−0.3 ± 7.8	−1.0 ± 8.2	−2.6 ± 10.9	0.876
ΔTK (°)	−5.6 ± 6.1	0.4 ± 6.7	1.8 ± 6.2	0.003*
ΔAPP (°)	9.4 ± 3.7	0.5 ± 3.9	−12.2 ± 6.3	<0.001*
ΔSVA (°)	8.4 ± 33.9	22.7 ± 37.8	25.1 ± 34.7	0.144
ΔSS (°)	0.2 ± 7.4	−1.2 ± 5.3	−3.0 ± 4.0	0.143

The Δ value defined as the difference between preoperative and 1‐year postoperative values.

APP, anterior pelvic plane; LL, lumbar lordosis; SS, sacral slope; SVA, sagittal vertical axis; TK, thoracic kyphosis.

* Statistically significant (*P* < 0.05).

### *Correlation between ΔAPP and Change in the Spinopelvic Parameters*

A weak negative correlation was observed between ΔAPP and ΔTK (*r* = −0.215, *P* = 0.032) (Table 4). A weak positive correlation was observed between ΔAPP and ΔSS (*r* = 0.237, *P* = 0.031) and ΔSVA (*r* = 0.242, *P* = 0.015).

**Table 4:** Correlation between ΔAPP and change in spinopelvic parameters

Variables	Correlation coefficient	*P*
ΔLL	−0.015	0.88
ΔTK	−0.215	0.032*
ΔSS	0.237	0.031*
ΔSVA	0.242	0.015*

The Δ value defined as the difference between preoperative and 1‐year postoperative values.

APP, anterior pelvic plane; LL, lumbar lordosis; SS, sacral slope; SVA, sagittal vertical axis; TK, thoracic kyphosis.

* Statistically significant (*P* < 0.05).

### *Correlation between ΔAPP and Preoperative Spinopelvic Parameters*

No significant correlation was identified between ΔAPP and most of the preoperative spinopelvic parameters. A significant, moderate negative correlation was identified between ΔAPP and preoperative APP (*r* = −0.418, *P* < 0.001) (Table 5).

**Table 5:** Correlation between ΔAPP and preoperative spinopelvic parameters

Variables	Correlation coefficient	*P*
Preoperative FA	−0.101	0.317
Preoperative LL	0.063	0.536
Preoperative TK	0.107	0.287
Preoperative APP	−0.418	<0.001*
Preoperative SVA	0.191	0.057
Preoperative SS	−0.093	0.355
Preoperative PI	−0.038	0.711
Preoperative PI‐LL	−0.112	0.269

The Δ value defined as the difference between preoperative and 1‐year postoperative values.

APP, anterior pelvic plane; FA, femoral angle; LL, lumbar lordosis; PI, pelvic incidence; SS, sacral slope; SVA, sagittal vertical axis; TK, thoracic kyphosis.

* Statistically significant (*P* < 0.05).

## Discussion

### *Change in Pelvic Tilt after Total Hip Arthroplasty*

In the current study, there was no significant change in PT 1 year after THA. Numerous studies have demonstrated that no significant difference has been observed between preoperative and postoperative PT^14^, ^15^, ^16^. Pelvic retroversion has generally been observed over the years after THA^19^, ^20^, ^21^. In addition, the chronological changes in PT in standing position were larger than the supine position at 5 years after THA^21^. More than 20% of PT demonstrated an increase in the risk of superior edge loading and posterior articular impingement^3^. Therefore, it is more important to evaluate the distribution of change in PT. A total of 81% of patients in this study had a change in PT less than 10°. This result is consistent with previous reports. The percentage of postoperative change in PT more than 10° that has been reported ranged from 14% to 17%^15^, ^16^, ^20^.

### *Posterior or Anterior Change in Pelvic Tilt after Total Hip Arthroplasty*

It is difficult to estimate anterior and posterior PT after THA. To clarify this issue, three groups were created based on postoperative changes in the APP. In the current study, pelvic retroversion (>5°) was more common (25%) than pelvic anteversion (16%). Ishida *et al*. demonstrated similar results, reporting that 13.4% of patients exhibited pelvic retroversion (>10°) and 3.4% of patients exhibited pelvic anteversion after THA^15^. In patients with preoperative marked pelvic anteversion, there was posterior change after THA, while patients with preoperative pelvic retroversion did not experience any significant anterior change. They concluded that there was a variation of anteversion or retroversion of the pelvis after THA. In contrast, the PA group had significant preoperative pelvic retroversion and the PR group had significant preoperative pelvic anteversion in this study. There was a significant correlation between preoperative APP and ΔAPP at 1‐year follow‐up.

### *Correlation between Spinopelvic Parameters and Pelvic Tilt after Total Hip Arthroplasty*

Preoperative pelvic tilt has been reported to be a predictive parameter for postoperative change in PT^15^, ^16^, ^17^. Lumbar spine degenerative conditions and vertebral fractures are also associated with the change in PT after THA^19^, ^22^. In the current study, preoperative spinopelvic parameters did not affect the change in PT after THA, except for preoperative PT. Sagittal spinopelvic alignment is important for maintaining standing posture^12^, ^13^. PI is a morphological parameter that is the sum of the sacral slope and PT. PI also regulates sagittal lumbar lordosis^23^. Previous reports did not focus on the relationship between PI and the change in PT after THA. Interestingly, preoperative PI and PI‐LL in the PA group were significantly smaller than in the PT group and the PR group in our study. In addition, the mean PI in the PA group was more than 10° smaller than in the normal Japanese population^24^. As a result, a negative PI‐LL mismatch was observed in the PA group. This phenomenon may be explained by patients with a small pelvic incidence having lower potential for compensation of the sagittal balance^9^. In patients with lumbar hyperlordosis, it was observed to be an alternative compensatory mechanism when pelvic retroversion reached its limit.

### *Limitations of this Study*

There are some limitations of the present study. First, preoperative and postoperative change in PT was assessed by a lateral view of the X‐ray only in standing position. In general, PT changes posteriorly from supine to standing position^20^, ^21^. Preoperative assessment with a dynamic evaluation in the standing and sitting or supine position reduces the risk of complications^7^, ^25^. Second, PT changes posteriorly over the years after THA^19^, ^20^, ^21^. In our study, the 1‐year follow‐up period may not have been long enough to assess the chronological change in PT. Third, complications such as articular impingement and dislocations were not investigated, and thus, the relationship between change in PT and complications was unclear. Fourth, we did not assess hip and knee contracture as well as lumbar degenerative conditions, which may affect the spinopelvic parameters. Finally, there were several types of implants included in this study.

In conclusion, preoperative PT was the only predictive factor associated with a marked anterior or posterior change in PT. Marked posterior pelvic tilt, small PI, and negative PI‐LL values were the factors that influenced pelvic anteversion after THA. A comprehensive assessment of spinopelvic parameters is essential for hip‐spine complex, including spinal alignment, pelvic alignment, and hip joint.
